# The role of *Streptomyces* to achieve the United Nations sustainable development goals. Burning questions in searching for new compounds

**DOI:** 10.1111/1751-7915.14541

**Published:** 2024-08-03

**Authors:** Miriam Rodríguez, Lorena Cuervo, Laura Prado‐Alonso, María Soledad González‐Moreno, Carlos Olano, Carmen Méndez

**Affiliations:** ^1^ Departamento de Biología Funcional e Instituto Universitario de Oncología del Principado de Asturias (I.U.O.P.A) Universidad de Oviedo Oviedo Spain; ^2^ Instituto de Investigación Sanitaria de Asturias (ISPA) Oviedo Spain

In the 21st century, the world is facing persistent global problems that have led to 193 countries to agree on the 17 Sustainable Development Goals (SDGs). The United Nations introduced these goals in 2015 to find solutions that could help end poverty, promote prosperity and protect the planet (United Nations, 2016a). In this brief perspective, we will discuss the potential role of *Streptomyces* in achieving those SDGs, focusing it in the current strategies applied for discovering novel compounds and in some of the problems that must be faced (Figure 1).

**FIGURE 1:**
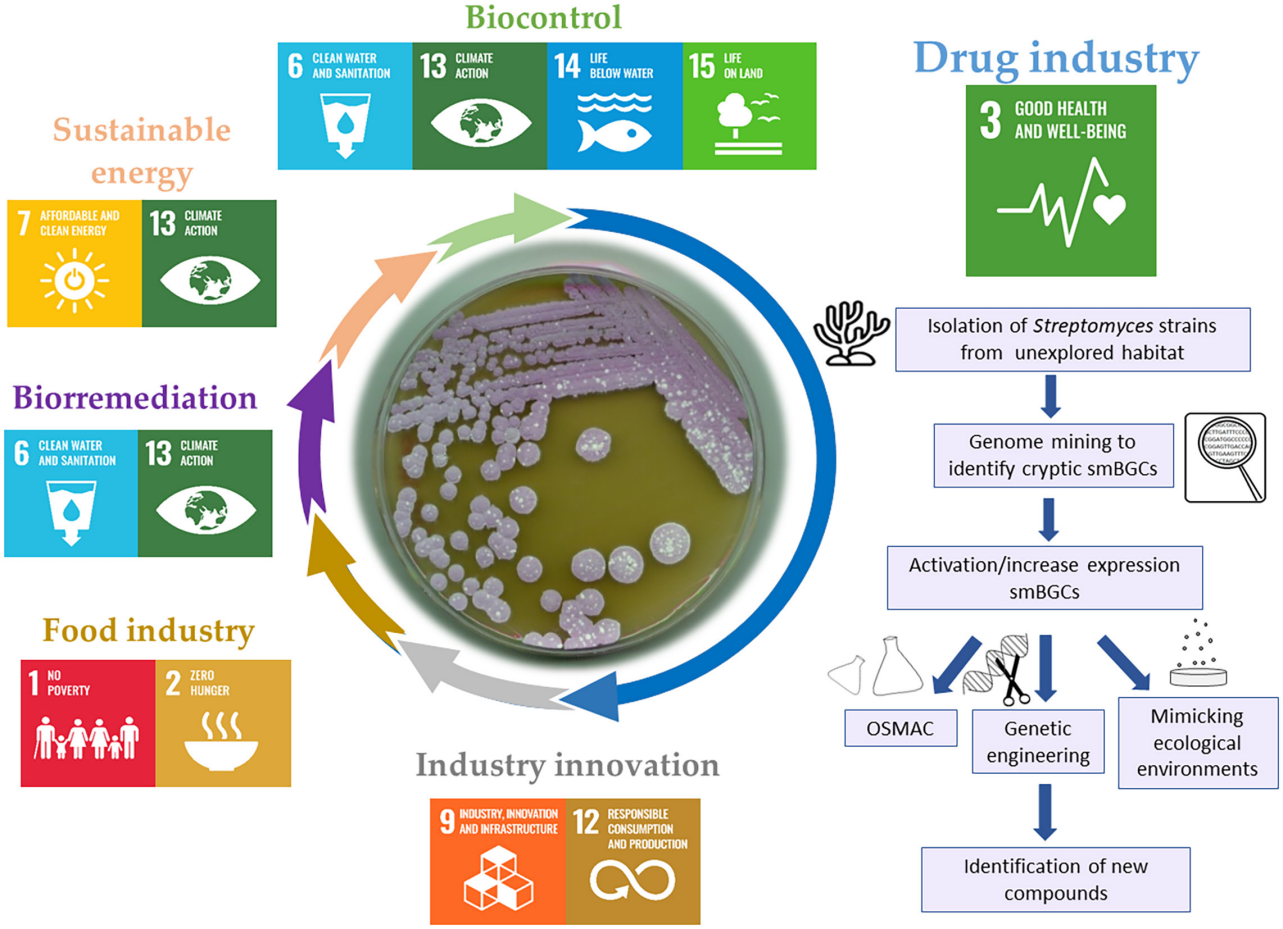
Strategies and applications of *Streptomyces* to achieve Sustainable Development Goals (modified from United Nations, 2016b).

Members of the genus *Streptomyces* are filamentous Gram‐positive bacteria belonging to the phylum Actinobacteria. They are ubiquitous microorganisms mainly found in soil but they can also inhabit other niches like seawater or deserts, or living associated with other organisms (Sivalingam et al., 2019). *Streptomyces* is mainly known for its ability to produce a wide array of bioactive secondary metabolites, which have several interesting applications in different fields (Alam et al., 2022; Demain & Sanchez, 2009; Donald et al., 2022).

One of the problems that most concern the United Nations is the existence of a growing demand for food in today's world (Food security information network, 2023). In this context, *Streptomyces* could play a relevant role in achieving SDG 2 (zero hunger, improved nutrition and sustainable agriculture) and SDG 1 (end poverty). *Streptomyces* produces several metabolites with significant commercial relevance in enhancing the nutritional value of human food and animal feed, such as vitamins like cobalamin (Rex et al., 2022). Additionally, there is an increasing need for enzymes in the global market (Grand View Research, 2023). *Streptomyces* due to its wide metabolic potential is used for the sustainable biotechnological production of a broad assortment of enzymes such as proteases, xylanases, amylases, lipases, keratinases, cellulases, dextranases and chitinases among others (Fernandes de Souza et al., 2022; Kumar et al., 2020). These enzymes have applications in several fields, and advantages not only in terms of energy consumption, stability, substrate specificity, purity or reaction efficiency but also in ecological and waste generation, thus contributing to the achievement of sustainable industrialization and innovation (SDG 9) and promoting responsible production and consumption (SDG 12). An example of enzymes with ecological applications is the degradation of lignocellulose and dye decolourization by detergent‐stable peroxidases and laccases (Cuebas‐Irizarry & Grunden, 2024). These enzymes can be potentially used to treat wastewater resulting from human activities like textile and paper industries, which cause environmental pollution and wastes that affect life below water (SDG 14). Another promising application of *Streptomyces* is its use to obtain energy from waste resources, what contributes to the pursuit of affordable and clean energy (SDG 7) and climate action (SDG 13). For instance, Muthusamy et al. (2019) were able to produce bioethanol from different agro‐residues using an *S. olivaceus* strain isolated from a mangrove sample. *Streptomyces* also contributes to the preservation of life on land (SDG 15) because they play a crucial role in sustainable agriculture and plant growth due to its participation in soil fertility (Hozzein et al., 2019). They contribute to phosphate and potassium biosolubilization, nitrogen supply to ecosystems, to stablish beneficial symbiosis with other rhizosphere microorganisms and to produce biocontrol agents such as phytohormones, antimicrobials, antifungals, pesticides, bioherbicides and insecticides (Boubekri et al., 2022; Li et al., 2021). Furthermore, the use of *Streptomyces* is considered an eco‐friendly and promising technology for bioremediation of contaminants like pesticides and heavy metals because they can degrade organic and inorganic compounds more efficiently and safely than chemical agents (Jagannathan et al., 2021).

Nevertheless, the greatest contribution throughout history of *Streptomyces* is as producer of bioactive compounds with applications in clinical, veterinary and agricultural fields, being the most important microbial source of bioactive compounds (Donald et al., 2022). In this context, this microorganism is an incredible force for achieving good health and well‐being (SDG 3). During the so‐called Golden Age of antibiotic discovery *Streptomyces* provided humanity with antibiotics, antifungal, anti‐parasitic, immunosuppressive agents and antitumor compounds, many of them currently used in clinical (Demain & Sanchez, 2009). Subsequently, limitations in classical search techniques and depletion of traditional habitats have led to the rediscovery of known compounds or the identification of a scarce number of compounds with new scaffolds. This, together with the high costs to develop new compounds for clinical and other uses, resulted in a drastic decline in the discovery of new drugs and the withdrawal of these research departments from some big pharma companies (Genilloud, 2017). Nevertheless, recent screening new approaches, such as the use of pathogenic bacteria conditionally expressing antisense RNA of essential genes, have led to discovering new antibiotics like platensimycin (Figure 2) (Genilloud, 2017). In addition, screening antibiotic active molecules for other activities identified a number of useful natural products (NPs), including some with antitumor activity such as actinomycin D (Figure 2) (Demain & Vaishnav, 2011). Subsequently, advances in ‐omics and sequencing methods, and the development of synthetic and genomic manipulation techniques in *Streptomyces* in the last decades, have led to the emergence of new strategies in drug discovery, such as genome mining and combinatorial biosynthesis. These approaches represent promising strategies for discovering novel bioactive NPs, in many cases with high structural diversity. Additionally, these strategies were improved when combined with the isolation of new *Streptomyces* strains from low explored environments, which produce structurally diverse bioactive NPs with potential clinical applications (Alam et al., 2022; Chen et al., 2021; Donald et al., 2022; Lacey & Rutledge, 2022; Qin et al., 2017; Quinn et al., 2020). Noteworthy, the antibacterial anti‐Gram positive chaxalactin (Figure 2) (Castro et al., 2018) produced by *Streptomyces leuwenhoeeki* from the Atacama desert; or cervimycins produced by *Streptomyces tendae* strain HKI 0179 from the ancient Italian cave Grotta dei Cervi, with antibacterial activity anti‐MRSA, anti‐VRE and anti‐ *S. aureus* EfS4 (Herold et al., 2005). Notably, in recent years, marine environments have been a prolific source of new NPs with a variety of bioactivities (Alves et al., 2018; Chen et al., 2021; Choudhary et al., 2017; Dharmaraj, 2010; Donald et al., 2022; Yang et al., 2020). Examples include the antibacterial anthracimycin B, produced by *Streptomyces cyaneofuscatus* M‐169 from the Cantabrian sea (Rodríguez et al., 2018); or the cytotoxic neo‐actinomycin A produced by *Streptomyces* sp. IMB094 from a marine sediment (Wang et al., 2017). Another unusual habitat where *Streptomyces* strains are found is in symbiotic associations with plants, fungi, vertebrates or invertebrate animals, both marine and terrestrial. In these associations, they appear to be a nutritional resource, or to play a protective role for the host against pathogens, parasites or predators, by producing antibiotic compounds (Barka et al., 2016; Batey et al., 2020; Chen et al., 2021; Donald et al., 2022; Qin et al., 2011; Seipke et al., 2012). In this context, it is worth highlighting the role played by some volatile compounds (VOCs) produced by *Streptomyces* such as geosmine, as an attractant for soil‐dwelling arthropods like springtails, to localize them as a food source. In turn, springtails facilitates the dispersal of *Streptomyces* spores to other niches by these arthropods (Becher et al., 2020). One of the most widespread example is the symbiotic relationship with insects. Thus, new antifungal compounds such as mycangimycins (Scott et al., 2008) or frontalamides A and B (Blodgett et al., 2010) have been isolated. Both are produced by symbiotic *Streptomyces* strains found in the southern pine beetle (SPB) *Dendroctonus frontalis*. Another example are the new formicamycins antibiotics (Figure 2) that have shown promising anti‐MRSA and VRE activities, which are produced by *Streptomyces formicae* KY5, isolated from African ants of the *Tetraponera* genus (Qin et al., 2017).

**FIGURE 2:**
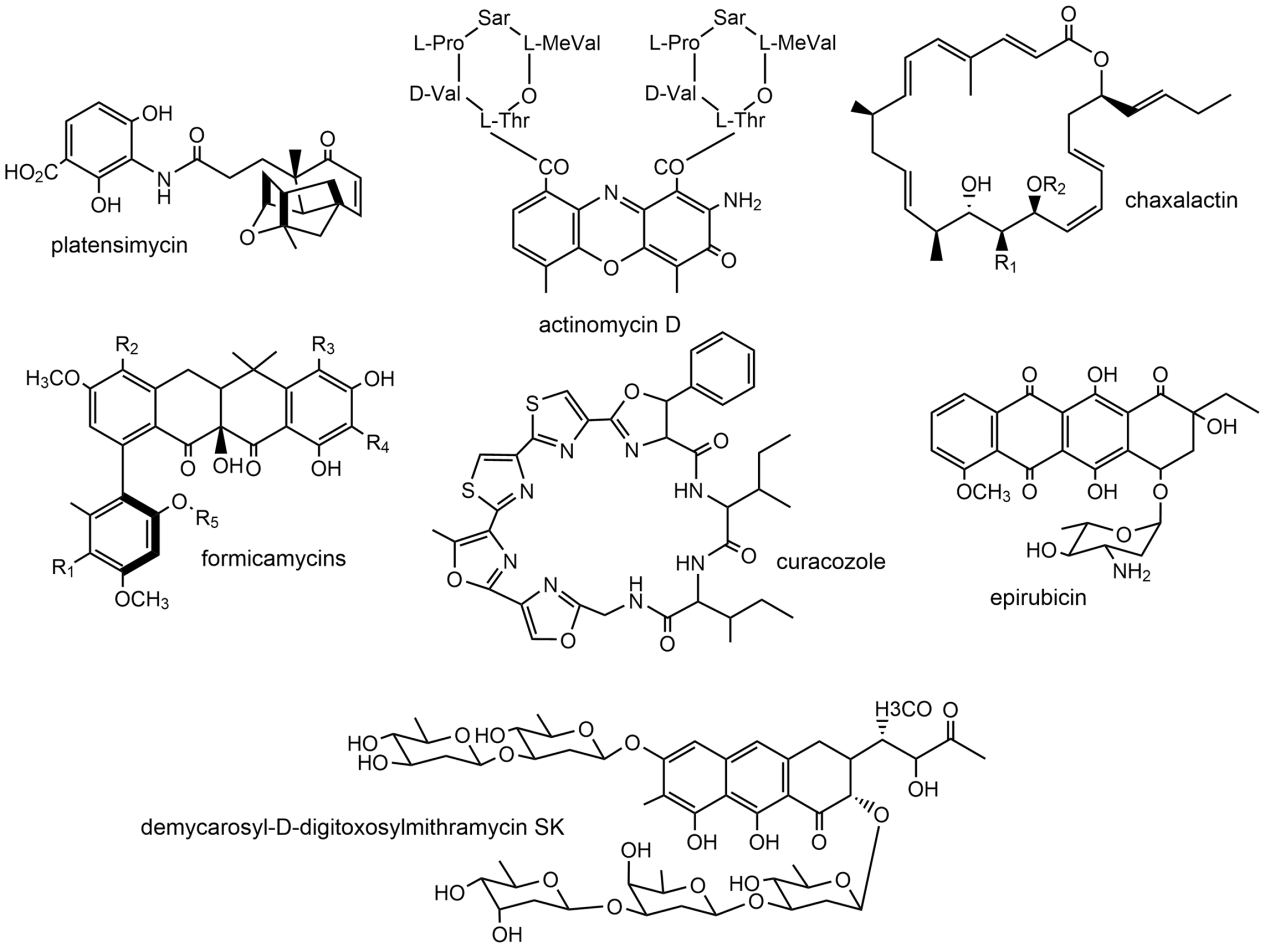
Chemical structures of some compounds produced by *Streptomyces* and identified using different strategies.

As it has been mentioned before, genome mining has become a useful tool for discovering natural products from the early 2000s (Baltz, 2021; Lee et al., 2020). It can be defined as the set of bioinformatics tools used to detect secondary metabolite biosynthesis gene clusters (smBGCs) and their possible functional and chemical interactions (Albarano et al., 2020). Genome mining has shown that each *Streptomyces* species possesses about 30 smBGCs, what has supported the hypothesis that most *Streptomyces* biodiversity is yet to be exploited for NPs discovery (Baltz, 2019; Belknap et al., 2020). In recent years, this strategy has enabled the identification of potentially new secondary metabolites encoded by smBGCs. For example, the antitumor chaxapeptin, identified by mining a *S. leuwenhoeeki* strain isolated from the Atacama desert (Castro et al., 2018); the antituberculous atratumycin, produced by *S. atratus* SCSIO ZH16 from the South China Sea (Sun et al., 2019); the new cytotoxic peptide curacozole (Figure 2), isolated from *Streptomyces curacoi* (Kaweewan et al., 2019); or largimycins, new leinamycin‐like compounds identified by mining *S*. *argillaceus* (Becerril et al., 2020). Nonetheless, despite some successful examples that can be found in the literature, the enormous diversity of smBGCs identified by genome mining is only partially translated to discovering new bioactive NPs, and identifying and characterizing compounds encoded by these predicted smBGCs still requires substantial laboratory work. Thus, the smBGC can be expressed or low‐expressed but the predicted encoded compounds is not detected under standard laboratory conditions (cryptic products), or the smBGC identified is not expressed and the product is unobserved (silent BGC with a cryptic product). All of these scenarios exemplify ‘Known Unknowns’ secondary metabolites (Hoskisson & Seipke, 2020). Therefore, a key issue for being successful using genome mining as an approach is to find strategies to turn on or to increase the expression of these silent or low expressed smBGCs. For this purpose, there are several genetic strategies that have been used like overexpression of positive regulators; inactivation of negative regulators; heterologous expression of the smBGC; or the insertion of a strong promoter upstream of BGC operons (Olano, García, et al., 2014). Other strategies to alleviate challenge of identifying the cryptic products are OSMAC (one strain of many compounds) (Pan et al., 2019); mimicking the ecological environment of the producer (Cuervo et al., 2022); redirecting precursors to the target biosynthesis pathway (Kallifidas et al., 2018); engineering global regulators (Cuervo et al., 2023); or ribosome engineering (Zhu et al., 2019). Nevertheless, we have to keep in mind that one of the major bottlenecks in drug discovery throughout history was the constant rediscovery of known compounds. From this perspective, some smart bioinformatics genome mining approaches can increase the chances to identifying unknown smBGCs encoding new compounds with potentially clinical applications. For example, several strategies have been used in recent years like mining for resistance genes (Culp et al., 2020), or for *Streptomyces* Antibiotic Regulatory Protein genes (Ye et al., 2023). Additionally, searching genes involved in the biosynthesis of unusual functional groups has also been used as an approach to select new smBGCs, such as targeting halogenases genes (Prado‐Alonso et al., 2022); DNA regions in Polyketide Synthases encoding the didomain DUF–SH specific for sulfur incorporation (Pan et al., 2017); C‐terminal thioester reductase (TR) domains and ϖ‐transaminases (Awodi et al., 2017); or piperazate synthase encoding genes (García‐Gutiérrez et al., 2024; Morgan et al., 2020).

Another important application of genome mining is as reservoir of genetic sets and devices for being used in combinatorial biosynthesis strategies. This method squeezes the maximum of synthetic biology techniques, by using different genetic engineering strategies to generate smBGCs with novel gene combinations. These would encode novel biosynthetic pathways that potentially could direct the biosynthesis of new natural products with different or improved properties. Combinatorial biosynthesis encompasses several strategies such as combination of native biosynthetic genes and genes from other smBGCs, expression of genes from other smBGCs into mutants blocked at specific biosynthetic steps, mutasynthesis based on the use of different biosynthetic precursors, or all of the above strategies combined to obtain new structural units (Olano et al., 2009). This method has been successfully used for the biosynthesis of new derivatives of a wide variety of compounds like terpenes (Tang et al., 2022), non ribosomal peptides (Ruijne & Kuipers, 2021), RiPPs (ribosomal synthesized and post‐translationally modified peptides) (Sardar & Schmidt, 2016), polyketides (Wang et al., 2022) or nucleosides (Niu et al., 2017). An interesting example was the generation of the new glycosylated analog demycarosyl‐3D‐β‐D‐digitoxosylmithramycin SK (Figure 2), derived from mithramycin (Núñez et al., 2012). Production of this compound was achieved by providing the capability to synthesize D‐digitoxose to a *S. argillaceus* strain mutated in a ketoreductase gene of the mithramycin BGC. This analog showed high antitumor activity and less toxicity than the parental compound, and among others, it is able to suppress EWS‐FLI1 activity suggesting a potential development in clinical (Osgood et al., 2016). Another example was the production of epirubicin (Figure 2), a less cardiotoxic doxorubicin derivative, which initially was produced by semisynthesis. A new method was designed for its production consisting in expressing *avrE* or *eryBIV* from the avermectin and erythromycin gene clusters into a *S. peucetius* doxorubicin non‐producer mutant (Demain & Vaishnav, 2011).

To summarize the current state of the art we can highlight a study carried out by Malmierca et al. (2018, 2020), which illustrates the combination of different chromatographic, genome mining, nutritional and combinatorial biosynthesis approaches, as an effective strategy for identifying new NPs. This research was conducted on *Streptomyces* strains isolated from symbiotic associations with leaf cutters ants of the *Attini* tribe. These ants maintain close association with *Streptomyces* that produce bioactive compounds, including antifungals and inhibitors *of Escovopsis weberi*, a parasitic microfungus of their mutualistic *Basidiomycete fungi* (Seipke et al., 2011). Malmierca et al. mined those *Streptomyces* genomes searching for smBGCs encoding glycosylated secondary metabolites, since many therapeutically relevant drugs contain sugar moieties (Salas & Méndez, 2007). By a combination of genome mining, PCR screening, metabolites dereplication, as well as genetic and nutritional approaches, they identified two novel compounds of the cervimycins family (sipanmycin A and B), and two novel members of the warkmycin family (Malmierca et al., 2018). Also, by combinatorial biosynthesis, expressing plasmids for the biosynthesis of deoxysugars into the sipanmycin producer *Streptomyces* CS149, they generated six different derivatives with altered glycosylation patterns (Malmierca et al., 2020).

Recent research, some of them summarized in this Editorial article suggest that *Streptomyces* remains the leading producer of bioactive compounds. This article emphasizes the contribution of these microorganisms to achieving SDG3. Moreover, recent years have seen the implementation of new methods that have revitalized the discovery of new natural products, accentuating the promising potential of *Streptomyces*. Even though, despite the discovery of new *Streptomyces* species and the identification of a large number of hypothetical smBGCs through genome mining research, only a small fraction of them have been characterized so far. This is mainly due to the limitations of these methods. For example, culturing new *Streptomyces* species from extreme environments under laboratory conditions is usually a challenge, as well as the heterologous expression of smBGCs, which is difficult and time‐consuming. Related to genome mining, one of the major issues is the quality of genomic sequences. Most of the sequences in public databases are in draft form. Although incomplete genome sequences may be adequate for assembling many small, non‐repetitive secondary metabolites smBGCs, they are unsuitable for large smBGCs like those encoding NRPS or type I PKS. These enzymes are typically involved in the biosynthesis of most compounds identified in drug discovery programs. Consequently, their encoding genes are often predicted to be scattered through several contigs, making challenging to identify the corresponding smBGCs (Baltz, 2021). Another drawback is that although powerful methods for the prediction of product structure from sequences exist, like antiSMASH (Blin et al., 2023), PRISM (Skinnider et al., 2020) or MIBiG (Terlouw et al., 2023) among others, they still have a relative high rate of false positives and generally are limited to identify smBGCs related to known ones. Moreover, once hypothetical smBGCs have been located, it remains a huge challenge to activate them. Additionally, predicting smBGCs is worthless without linking them to their final product and/or expected biological activities (Lee et al., 2020; Olano, Méndez, & Salas, 2014; Ren et al., 2017). On the other hand, it is interesting to note that although the new strategies developed in *Streptomyces* have shown the potential to discover pharmaceutically important drugs, they have not been successfully integrated into pharmaceutical company pipelines. This could be due to several factors, such as low throughput fermentation, challenges in natural product optimization, and declining return on investment (Baltz, 2021; Ward & Allenby, 2018).

## AUTHOR CONTRIBUTIONS

**Miriam Rodríguez:** Writing – original draft; writing – review and editing. **Lorena Cuervo:** Writing – original draft. **Laura Prado‐Alonso:** Writing – original draft. **María Soledad González‐Moreno:** Writing – original draft. **Carlos Olano:** Writing – review and editing; funding acquisition. **Carmen Méndez:** Writing – review and editing; funding acquisition.

## CONFLICT OF INTEREST STATEMENT

The authors declare no competing financial interest.
